# Infants’ Selective Visual Attention Is Dependent on Maternal Affect and Emotional Context

**DOI:** 10.3389/fpsyg.2021.700272

**Published:** 2021-09-16

**Authors:** Joshua Juvrud, Sara A. Haas, Nathan A. Fox, Gustaf Gredebäck

**Affiliations:** ^1^Department of Psychology, Uppsala Child and Baby Lab, Uppsala University, Uppsala, Sweden; ^2^Department of Psychology, University of Maryland, College Park, MD, United States

**Keywords:** selective attention, negative affect, face emotion, emotional context, infancy, maternal

## Abstract

Development of selective attention during the first year of life is critical to cognitive and socio-emotional skills. It is also a period that the average child’s interactions with their mother dominate their social environment. This study examined how maternal negative affect and an emotion face prime (mother/stranger) jointly effect selective visual attention. Results from linear mixed-effects modeling showed that 9-month olds (*N*=70) were faster to find a visual search target after viewing a fearful face (regardless of familiarity) or their mother’s angry face. For mothers with high negative affect, infants’ attention was further impacted by fearful faces, resulting in faster search times. Face emotion interacted with mother’s negative affect, demonstrating a capacity to influence what infants attend in their environment.

## Introduction

From birth, infants actively gather information from their environment through visual exploration (Amso et al., 2010). This ability is supported by an early developing attentional mechanism, selective attention, which serves as an information filter, determining which of the competing visual stimuli is given access to further processing for perception, memory, and influencing subsequent behavior (Craik et al., 1996; Markant and Amso, 2013). Goal-directed behavior is supported by the visual information that is prioritized and given current contextual relevancy, while representations that are less important and distracting are weakened (Desimone and Duncan, 1995). Objects in the environment that are prioritized by the attentional system can depend on features of the stimuli themselves, such as saliency (bottom-up perspectives on attention; Theeuwes et al., 2000), but also through certain objects having significantly more relevancy based on the task at hand or prior experiences (top-down, e.g., Desimone, 1996; Logan, 2002; Kristjánsson, 2006; Kristjánsson and Campana, 2010; Chun and Johnson, 2011; Awh et al., 2012). An adaptive and flexible selective attention system is critical to learning (Walther et al., 2005; Amso and Johnson, 2006; Bhatt and Quinn, 2011; Markant and Amso, 2013), development of executive functions (Rueda et al., 2005; Wass et al., 2011; Johansson et al., 2015; Yu and Smith, 2016; Veer et al., 2017), and socio-cognitive abilities (Pollak and Sinha, 2002; Masten and Obradovic, 2008).

**Figure 1:**
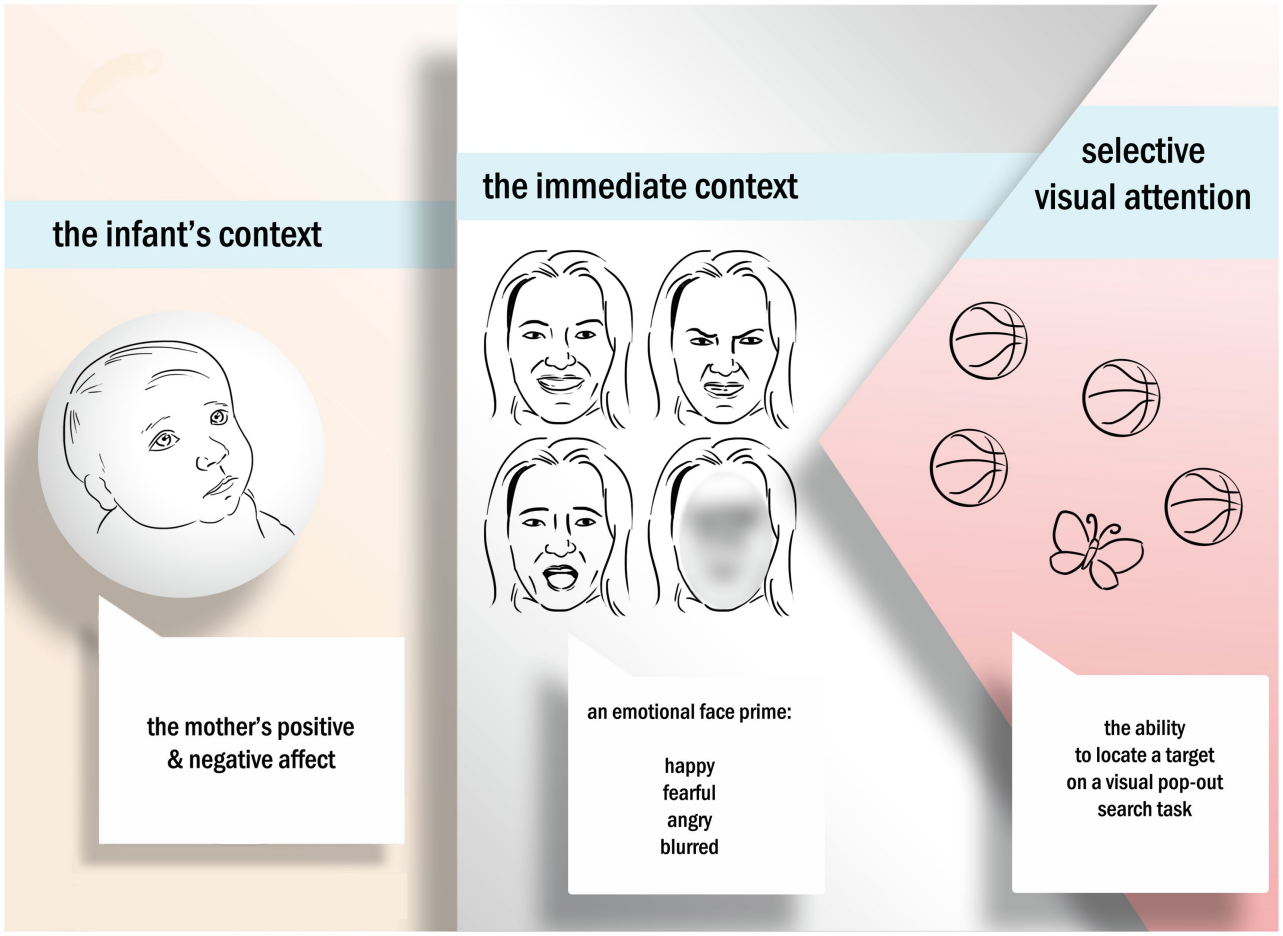
The current study examines how infants within an integrated system of caregivers with high levels of negative affect, and are exposed to negative emotional expressions, are differentially impacted in their ability to selectively encode and learn from their environment through selective visual attention.

The attentional system is adaptive, changing depending on context and experiences (Colombo, 2001). We know that infants’ sensitivity to emotional content within the immediate context, such as exposure to an emotional face, results in a change to infants’ visual processing efficiency (Davis et al., 2011). For example, Montague and Walker-Andrews (2001) showed a differential sensitivity to emotional expressions in a dyadic context with 4-month-old infants. In a peek-a-boo paradigm, infants showed differential responses to distinct emotional expressions based on the familiarity of the context. That is, when infants were exposed to emotional expressions from their mother in a familiar context (peek-a-boo), they showed early sensitivity to emotion. A large body of work also shows that the maternal context provided by the infant’s mother (Feinman, 1982; Feinman and Lewis, 1983; Hornik et al., 1987; De Haan et al., 2004), and the kinds of visual experiences that infants have with their mothers during early infancy, can shape attentional mechanisms and abilities. Visual experiences, such as affective communication that mothers have with their infants, can aid infants in regulating their actions and attention (Hornik et al., 1987; Tronick, 1989). Affective communication can include displays of emotion, and indeed, infants show a sensitivity to emotional faces (Thompson-Booth et al., 2014; Porto et al., 2020) and an increase in attentional resources within the environment. In other words, the adaptive nature of the attentional system means that the infants’ immediate context, in particular the emotional content and emotional content provided by the mother, may influence how infants’ views their world (see Figure 1). Independently, both the immediate context (emotional content an infant is immediately exposed to, such as facial expressions) and the infant’s general context (such as affective states the mother might possess over time) have shown potential effects on selective visual attention. Yet, there is still a gap in understanding the link between the infant’s context, the immediate context, and the subsequent joint effects of these on selective visual attention.

## Exposure To Facial Expressions on Selective Attention

We know from many studies that perceived threat (threat detection) influences the allocation of selective attention resources by modulating the ability to rapidly detect and respond to potentially threatening stimuli (Phelps et al., 2006; Becker, 2009; Pereira et al., 2010; Olatunji et al., 2011; Aktar et al., 2018). For instance, exposure to an immediate emotional stimulus within the environment, such as a fearful facial expression, can facilitate heightened attention capture. Fearful faces, in particular, elicit increased activity in the amygdala, communicating to the individual the presence of potentially threatening stimuli, and affecting attentional resources (Williams et al., 2005; Bishop, 2008). We know that an attunement to threat-cuing facial expressions begins developing by 7months in infants—by this age, some infants are able to differentiate between emotional expressions and begin attending more to negative emotions (Nelson and Dolgin, 1985; Kotsoni et al., 2001; Balconi and Pozzoli, 2003; De Haan et al., 2004; Peltola et al., 2009).

Emotional faces of fear and anger (commonly labeled as negative emotions) have been shown to differentially impact visual attention to the environment in adults and result in a more generalized increase in processing efficiency (Davis et al., 2011). These emotional facial expressions, in particular, serve as an important cues for attending to the immediate environment. While both anger and fearful are considered “threat-related” cues, Davis and colleagues showed that fearful faces communicate an increased probability of a threat, whereas angry expressions embody a certain and direct threat. Because fearful faces do not signal a certainty of threat, a fearful face will facilitate the processing of the environment to gather information to disambiguate the threat, while angry faces direct attention toward the angry individual (Davis et al., 2011). The findings by Davis et al. (2011) in adults have yet to be replicated with infants. However, infants rarely see faces without context, and there is evidence that there are changes in infants’ attentional processes as a result of emotional faces, which can vary depending on early experiences (Pollak et al., 2000). Therefore, we expect that threatening faces similarly modulate infants’ selective visual attention.

A state of negative arousal in the immediate context, the here and now, directly influences the efficiency of visual attention in both infants (de Barbaro et al., 2017) and adults (Davis et al., 2011). Negative arousal can be primed with an emotional facial expression, such as threat-signaling emotions like fear and anger, resulting in different patterns of environmental scanning (Becker, 2009; Haas et al., 2017). It has even been demonstrated with adults that threat-relevant faces result in a more generalized increase in processing efficiency, even toward non-emotional stimuli (Becker, 2009). In infancy, a large body of work has been focused on how infants’ visual attention selectively processes emotional faces, such as how quickly an infant disengages from a fearful face (Peltola et al., 2009; Kataja et al., 2019). But what happens after an infant sees an emotional face? Research has yet to fully explore how exposure to negative emotional facial expressions impacts subsequent visual attention to the environment.

## The Role of Maternal Care on Selective Attention

The selective attention system has strong biological underpinnings (Colombo, 2001), but it is also strongly influenced by interactions between the child and his or her environment (Calkins, 2011; Colombo and Salley, 2015; Swingler et al., 2015). The selective attention system is particularly sensitive to regularities in the environment. In the first year of life, the primary caregiver of the child is of particular importance as source of regular information for infants, and for the majority of infants at this age, the primary caregiver is the mother (Family Caregiver Alliance, 2019). During this phase of growth and learning, when maternal care dominates the child’s social environment, maternal behavior and affect can significantly contribute to individual differences in the changing neural systems underlying visual selective attention (Luthar et al., 2000; Cicchetti and Dawson, 2002; Posner et al., 2014). The early neuroplasticity and adaptability of the neurocognitive system underlying selective attention means that there are variations depending on the child’s environmental contexts and experiences (Chelazzi et al., 2013; Swingler et al., 2017). Therefore, during the early development of selective attention processes, such modulations of selective attention influence how infants and children perceive and interpret the world around them.

The kinds of visual experiences that infants have with their mothers during early infancy can shape attentional mechanisms and abilities, such as visual preferences for emotional face expressions (Striano et al., 2002), face emotion perception (Gredebäck et al., 2012), perceptual narrowing of faces (Rennels et al., 2017), and allocation of attention toward internal features of a face (Juvrud et al., 2019). Increased exposure to threating facial emotions has been shown to result in attention bias to threat cues and changes in neurocognitive systems underlying attention (Mogg et al., 1994; Pollak et al., 1998; Pine et al., 2005; Romens and Pollak, 2012). In particular, low levels of positive emotional expression in mothers have been associated with higher rates of sensitivity to maternal negative emotion shifts (Hatzinikolaou and Murray, 2010), instability in mother-child interactions (Reck et al., 2004), and later difficulties in labeling basic emotional expressions (Meiser et al., 2015).

The subjective wellbeing of the mother (maternal affect; Diener and Emmons, 1984; Karazsia and Wildman, 2009), and in particular the mother’s negative affect, is an important cue for infants to use when making own appraisals of events and regulating behavior (Feinman, 1982; Feinman and Lewis, 1983. Infants tend to respond more immediately to negative than positive emotional maternal signals (Reschke et al., 2017). Moreover, long-term exposure to a mother’s affective state can modulate an infant’s sensitivity to affective stimuli in his or her environment (Choi et al., 2017; Swingler et al., 2017). For instance, maternal affect can directly influence an infant’s own responses by modifying the emotional climate through direct emotional resonance and contagion mechanisms (Hornik et al., 1987). Maternal affect may influence infants’ appraisal of an event, result in a mood modification, or facilitate or inhibit infants’ responses.

Importantly, longitudinal measures of maternal negative affect have been shown to have lasting effects on children’s development (Weinberg and Tronick, 1998, Eisenberg et al., 2001). In a longitudinal study, Brooker et al. (2016) showed that maternal negative affect over the course of early infancy moderated later levels of cortisol and long-term coping capacities in children at 7years of age. It would appear that prolonged exposure to negative affect during the infancy period has significant consequences for stress physiology (Lawler et al., 2019), the development of regulatory skills [e.g., coping skills; (Davis et al., 2020)], and a greater risk for anxiety problems (Degnan et al., 2010). Related, refugee children (6–18years) whose mothers suffer from large amounts of war related posttraumatic stress symptoms (PTSS) are worse at identifying emotional facial expressions than children are similar contexts but with mothers that are less affected by PTSS. Another study has shown that infants with mothers that have symptoms of anxiety have a specific visual sensitivity to negative emotional expressions (Kataja et al., 2019). Based on these studies, we argue that there are multiple ways in which the maternal context might affect foundational (or supporting) information gathering processes, such as selective attention. Therefore, we believe that further examining infants sensitivity to maternal negative affect in relation to visual attention is warranted and may provide additional insights into the dynamic relationships between infants, caregivers, and the environment.

## Current Study

The rapid development of selective attention during the second half of the first year of life is important for later childhood cognitive competencies and socio-emotional skills (e.g., Bhatt and Quinn, 2011). It is also a period in which the average child’s interactions with their mother dominate their social environment (Family Caregiver Alliance, 2019). Both maternal affect from the infant’s general context and emotion stimuli in the immediate context influence and shape visual selective attention mechanisms but have largely been explored independently. Here, we conceptualize maternal negative affect as a maternal mood disturbance and an expression of feelings, such as anger, contempt, shame, fear, and depression (Hanley et al., 2014). High maternal negative affect may not necessarily include an increased exposure to negative facial expressions, but it is possible they are related. It is still unclear to what extent exposure to maternal affect and immediate emotional stimuli in the environment, such as actual immediate facial expressions, interact and jointly effect selective attention during the first year of life, a period of paramount importance for neural development and learning (Markant and Amso, 2013). It is imperative to bring these two domains together in order to understand how infants who develop within an integrated system of mothers with high levels of negative affect, and who may also be more frequently exposed to negative emotional expressions in their environment, are differentially (and perhaps sub-optimally) impacted in their ability to selectively encode information.

Using an integrative approach, the aim of this study is to examine how selective visual attention to non-emotional stimuli is jointly impacted by the interaction between maternal negative affect and emotion face primes in the immediate context. We hypothesized that, given the evidence for the importance of the mother’s emotional expressions during infancy and that the mother serves as a particularly important source of information about the infants’ world, infants’ selective visual attention to their environment will be differentially impacted after viewing different emotional expressions of their mother. To test this, we measured infants’ performance on a non-emotionally valenced visual search task after being primed with an emotional face (immediate context; happy, fearful, anger, and blurred). We used latency on the visual search task as a measure of search vigilance. In the current study, the latency speed is a measure of attentional control. Slower latencies mean there is increased attentional control (the mechanism) and is represented through vigilance (the outcome). This means that slower latencies indicate increased vigilance, and the underlying mechanism for this increased vigilance is increased attentional control. To examine further the significance of the mother in the infant’s general developmental context, the emotional face shown to the infants was either that of a stranger or of their own mother.

Prior work has shown that, apart from emotional face expressions, the subjective emotional state of the mother can have various consequences for both infants’ direct emotional resonance and subsequent visual attention (Brooker et al., 2016). Therefore, we also examined infants’ performance on a visual search task in relation to the mothers’ levels of maternal affect. We predicted that the negative affect of the mother would interact with exposure to various emotional facial expressions and result in modulations of infants’ selective visual attention to their environment.

Prior work has demonstrated that increased sensitivity to particular emotional face expressions, such as anger and fear, emerges by 5months of age and is well established between 7 and 9months of age (Xie et al., 2019). The current study was conducted with families in Sweden, a country with generous laws for maternity and paternity leave that often result in fathers transitioning to the primary caregiver after the first year (Rennels et al., 2017). Therefore, the age of 9months provides us with a unique and narrow age window where emotion recognition and processing abilities are further developed, infants have sufficient experience with their mothers’ various affective states, and the mother is still the primary caregiver. A visual pop-out search task was chosen as an age-appropriate measure for visual attention (Adler and Orpecio, 2006). The number of distracters was also manipulated as a way to increase the difficulty, with more distractors requiring more attentional resources, and thus, performance potentially being more impacted by processing efficiency (Wolfe, 1994).

## Materials and Methods

### Participants

Seventy full-term 9-month-old infants (40 females; mean age = 8months and 27days, *SD*=9.55days) participated in the study. Another 10 infants were tested but excluded from the analysis due to technical error (*n*=5), infant inattentiveness/fussiness (*n*=4), and born premature (*n*=1). Infants were recruited from parents who had previously volunteered to participate in infant and child research.

To determine sample size and calculate power, we used the 16 total unique combinations of repeated conditions (familiarity, set size, and emotion), and assumed our repeated measurements have a high degree of correlation of at least 0.5, to achieve power of 0.95 and a moderate effect size of *η*^2^>0.06. We would therefore need at least 32 participants. A sample size of 40 infants, using the most conservative power assessment (64 trial conditions), results in a power of 0.88 to detect moderate effects and requires a critical *F* value of 3.30 or above to be confident in the effects.

Parents of infants provided informed consent and received gift voucher worth approximately 10€ for participating. The study was conducted in accordance with the standards specified in the 1964 Declaration of Helsinki and approved by the local Ethics Committee.

### Apparatus

We used E-Prime Professional 2.0 with Tobii Extensions to present stimuli on a 33.7×27cm (1,280×1,024 resolution) monitor and a Tobii T120 near infrared eye tracker (0.15° precision, 0.4° accuracy, and 60Hz sampling rate) to measure infants’ eye movements. Infants sat on their parent’s lap ~60cm from the monitor (0.022×0.023 visual degrees per pixel). A curtain separated the infant and parent from the researcher.

### Stimuli

#### Face Stimuli

**Figure 2:**
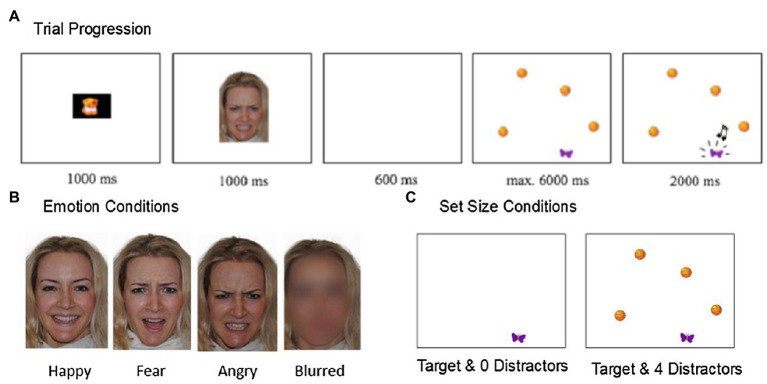
(A) Trial Progression (B) Emotion Conditions (C) Set Size conditions.

Emotional facial expressions consisted of color images of adult female faces (~11×15cm, 4.2×6.5 visual degrees) with the emotional expressions happy, fearful, and angry, as well as an additional blurred neutral face (see Figure 2). The reason for blurring the photographs was to provide a neutral comparison. Using a neutral expression can often result in perceiving the face as negative (Lee et al., 2008) and scrambling, the face results in the face losing structure and creating dissimilar areas of luminance, saliency of face regions, and changes due to contrast. Another reason the faces were blurred (as opposed to, e.g., scrambled) was for ecological validity. In real life situations, facial emotional expressions are often identified based on partial or suboptimal information and may generalize better to their ability in real life situations (see Forslund et al., 2017; Gredebäck, et al., 2021).

The faces used were either that of the infant’s own mother or a stranger (pseudo-randomized; the stranger’s face was always that of the previously tested infant’s mother). Photographs of the mothers were taken in a room with standardized lighting prior to testing and included the hair and neck with a white sheet covering the shoulders. All mothers were asked to remove makeup, jewelry, and glasses. To standardize emotional expressions, the mothers used a mirror and example photos of people expressing the different emotions from the Karolinska Directory of Emotional Faces (Lundqvist et al., 1998). The stimulus faces were shown with all facial features displayed (i.e., both internal and external facial features) and were standardized for brightness, contrast, color, and size using Adobe Photoshop CS5. A Gaussian blur filter was applied to the neutral face (22 pixels) so that no features of the face were visible (Gilad-Gutnick et al., 2012).

#### Visual Search Task

The visual search task was adapted for infants based on the procedure from Haas et al. (2017). Visual search targets consisted of static color cartoon objects (hat, butterfly, apple, cheese, pear, and sweater; 3×2cm; 1.2×1.0 visual degrees). Set sizes were either zero or four distractors (set size 1 and set size 5, respectively). Distractor pairings included as: hat and banana, butterfly and basketball, apple and car, cheese and flower, pear and bow, and sweater and planet (3×2cm; 1.2×1.0 visual degrees). Since the design was a pop-out search task, all distractors were the same image (e.g., banana) with the target being a distinctly different image (e.g., hat). Within and across infants, target location during visual search and the number and location of distractors was counterbalanced. All targets were of equal distance from the center of the screen (10cm) and not overlapping with the location of the previously presented face stimulus. Reward stimuli were presented when the infant fixated on a target and consisted of a short animation of the target moving up and down accompanied by a chime sound.

#### The Positive and Negative Affect Schedule

The self-assessment positive and negative affect schedule PANAS (Watson et al., 1988) was translated to Swedish and used to assess negative affect of participating mothers. Based on prior work (Hornik et al., 1987), the positive affect scale was not used in the current study as we did not expect any significant interactions with infants’ selective visual attention. The PANAS consists of a list of 10 emotional descriptions of negative states (*desperate, upset, feeling guilty, afraid, hostile, legal, shameful, nervous, angry/fearful, and frightened*). Mothers responded how much the emotional state accurately described themselves within the past week using a five-point Likert scale, ranging from “Not at all” to “Very much.” Test-retest reliability with a one-week interval for the negative affect scale has been found to be 0.79 and the Cronbach’s *α* is 0.84–0.87. Factorial validity has been shown to be good with convergent correlations between 0.89 and 0.95 and discriminatory correlations between −0.02 and 0.18 (Watson et al., 1988; Crawford and Henry, 2004).

### Procedure

An experimenter first obtained informed consent and photographed the mother’s facial expressions while another experimenter and/or parent accompanied the child in a separate waiting room. While the experimenter standardized the photographs and uploaded them to the experiment, the mother completed the affect measures, followed by the infant participating in the eye-tracking task. During the eye-tracking task, mothers were seated on a chair and infants sat on their parent’s lap in facing toward the stimulus monitor. During testing, mothers were instructed to limit their interactions with their infant to not direct their attention toward the screen and the mothers wore opaque eyeglasses to avoid unintentionally influencing the infant’s looking behavior. The experiment was started when all five points were successfully calibrated, which consisted of drawing the infants’ attention to the calibration stimuli at five 3×3 points on the screen. The calibration procedure was repeated for the missing calibration points (for more information about the calibration procedure, see Gredebäck et al., 2009).

Infants then viewed an attention grabber followed by a random emotional face from either the mother or stranger presented for 1,000ms. Face emotion and familiarity were randomized across trials. Infants then saw a blank screen (600ms) followed by the visual search task. A short reward animation was gaze-contingent and automatically played (2,000ms) when the infant fixated anywhere within the target image (100ms; Wass et al., 2013). Trials automatically advanced without a reward if the infant failed to fixate on the target within 6s. An attention grabber centered the infants’ gaze on the screen between each trial and the next trial only began when the infant’s gaze was fixated on the center of the screen. The infants viewed a total of 64 trials (32 per set size and eight per emotion) and the task was approximately 10min long, with the total visit lasting approximately 45min. Pilot testing enabled us to determine appropriate lengths for the face presentation, visual search trial lengths, and reward length that were suitable for infants. Parental-reported infant temperament data were also collected (see Supplementary Materials); however, given a high correlation and concerns of reliability of maternal report of infant behavior, only maternal self-report on the PANAS was examined in the current study. The decision to rely on the PANAS rather than temperament was due to open questions in the literature regarding reliability and validity of parent-reported temperament questionnaires (Seifer, 2002).

### Data Analysis

#### Areas of Interest

The analysis of eye-tracking latencies was performed in the open source analysis program TimeStudio version 3.03 (Nyström et al., 2016) in Matlab (version R2018b). Areas of interests (AOIs) were defined around each target and distracter image (3×2cm). Fixations were included in the final analysis if the infant fixated on the target for a minimum of 100ms, but less than the trial duration of 6,000ms. The design was gaze-contingent, meaning that a fixation within the AOI of the target image would automatically record the trial as a success and advance the trial to the reward animation. The actual data, analysis, settings, and source code used for analyzing the data can be downloaded with uwid ts-674-f5e from the TimeStudio interface.

#### Statistical Analyses

We utilized linear mixed-effects modeling (LMM) using SPSS 20 to handle both repeated trial-level data, as well as repeated measures of the visual search condition. Linear mixed-effects modeling was determined to be more appropriate than repeated measures RM-ANOVA, as LMM is a robust modeling framework for the analysis of repeated trial-level data when there are variable contributions from each participant (e.g., missing data; McCulloch, 2008). Thus, instead of determining an arbitrary cutoff for excluding infants with low trial data, we were able to consider all available data. On average, infants contributed 36 trials (*SD*=10.65, *Range* = 6–54 trials). If an infants failed to fixate within the 6s trial, that trial was omitted from the final analyses. In addition to our main analysis, we conducted a LMM to determine whether there were differences in the number of usable trials as a function of emotion, set size, or familiarity conditions (as well as interactions of all conditions). There were no significant differences across any of the conditions (or interactions) other than a main effect set size [*F*(1, 824.33)=773.59, *p*<0.0001]. Specifically, across the sample, infants contributed significantly more successful trials for the easier condition with no distractors, than the condition with four distractors. Table 1 shows the average usable trials for each of the conditions. This main effect was expected, as greater difficulty on visual search trials is associated with lower visual search accuracy consistently across children and adults (Wolfe, 1994).

**Table 1:** Mean usable trials across conditions.

	Trials (*SD*)	
**Total usable trials (Max 64; Range 6:54)**	36.03 (10.65)	
**Set Size (Max 32 per condition)**	**Trials (*SD*)**	
Set Size 0	24.67 (6.58)	
Set Size 5	11.52 (5.36)	
**Familiarity (Max 32 per condition)**	**Trials (*SD*)**	
Familiarity 0	18.04 (5.65)	
Familiarity 1	17.98 (5.57)	
**Emotion (Max 16 per condition)**	**Trials (*SD*)**	
Angry	9.02 (3.14)	
Fear	8.78 (2.95)	
Happy	9.08 (3.07)	
Blurred	9.12 (3.00)	
**Emotion×Set Size (Max 8 per condition)**	**Set Size 0 Trials (*SD*)**	**Set Size 0 Trials (*SD*)**
Angry	6.39 (1.68)	3.07 (1.48)
Fear	6.15 (1.81)	3.06 (1.45)
Happy	6.04 (2.02)	3.27 (1.62)
Blurred	6.25 (1.89)	3.14 (1.68)

To handle variations in infant data points across the sample, trial-level data for each infant, across the entire infant visual search task, were included in the model. The AR1 covariance structure assumes that correlations between repeated measures (e.g., sequential trials) closer together are more highly correlated, while those further apart have lower correlations (e.g., Trial 1 vs. Trial 64; see Wolfinger, 1993 for further explanation). Thus, the AR1 covariance structure was deemed the most appropriate given that the correlation between subsequent trials will be highly correlated, while trials further apart in time during the task will have smaller correlations. The restricted maximum likelihood criterion was used as it is a less biased estimation than traditional maximum likelihood.

We computed a LMM with participant as a random effect to allow for individual variability in baseline eye movement reaction times. The remaining predictor variable of infants’ context was maternal negative affect (PANAS), defined as a continuous fixed effect in our model. The predictor variables of infants’ immediate context were as: Emotion (angry, fearful, happy, and blurred) as a categorical fixed effect, coded familiarity (mother = 0 and stranger = 1), and set size type (no distractors = 0 and 4 distractors = 1) as continuous fixed effects. While the set size was coded in the data as zero or one, the included figures and discussion of the results refer to these as set sizes 1 and 5, respectively. The outcome measure of visual selective attention defined in this model was latency in milliseconds to locate the target in the visual search. Both parameter estimates as well as follow-up multiple comparisons with Bonferroni adjustments were examined to determine significant main effects and interactions. To ensure there was no relation between the mothers’ facial emotions and negative affect, we confirmed that the negative affect scores of the mothers were normally distributed and checked that there was no significant interaction between negative affect and familiarity.

We intentionally did not manipulate or standardize the facial emotions of the mother. The reason being is that this provides a more ecologically valid test of an infant’s familiarity with their mother’s emotional expressions if it is the mother’s natural expressions. Because this was a variable of interest in our analyses (familiarity), ecological validity was important for our interpretation of the findings. In addition, the pairings were pseudo-random (previous participant’s mother was the stranger face for the next participant) and the negative affect of the mothers was normally distributed.

For illustrating data in figures, we computed reaction times as difference scores between set size 5 and set size 1.

## Results

### Main Effects

There were several main effects, as well as superseding interactions. Model results indicated a significant effect of set size [*F*(1, 2431.458)=2321.264, *p*<0.001], such that the speed to find the target was faster for set size 1 [*t*(125.53)=2.97, *p*<0.001]. There was also a main effect of emotion *F*(3, 2405.975)=5.456, *p*<0.001 such that overall, latency to detect the target after exposure to a happy face was significantly longer than after exposure to a blurred prime [*t*(125.53)=2.97, *p*<0.001], angry prime [*t*(125.53)=2.97, *p*<0.001], or fearful face prime [*t*(125.53)=2.97, *p*<0.001]. See Supplementary Table S1 for the full model and Supplementary Table S2 for parameter estimates.

### Interactions

**Figure 3:**
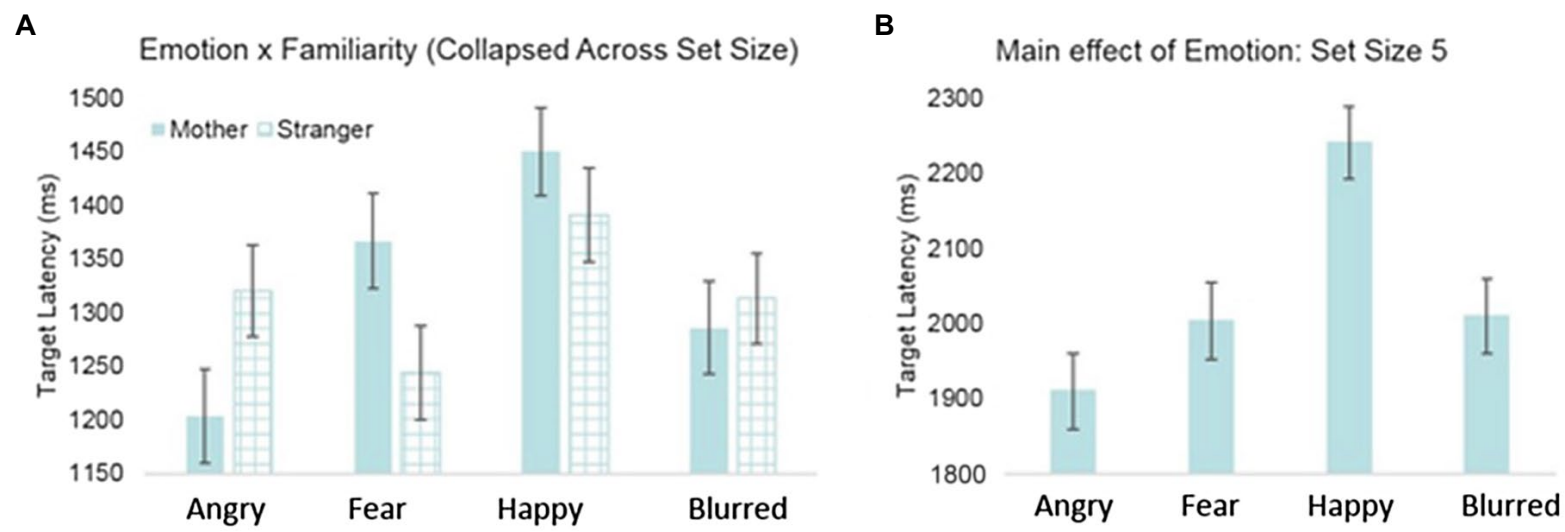
Latency to detect target by (A) emotion and face familiarity and (B) emotion for set size 5.

Table 2 displays the significant interactions (see Supplementary Materials for full model statistics). Significant effects of infants’ immediate context were found with a significant two-way interaction of emotion and familiarity, such that the effects of the fearful and angry primes had opposite effects on attention depending on whether the primes were of the mothers or a stranger’s face. *Post-hoc* analyses for the angry emotion condition revealed a trend that infants had shorter latencies to find the targets when they were primed with a mother’s angry face, compared to a stranger’s, *F*(1, 2405.33)=3.801, *p*=0.051 (see Figure 3A). The opposite was true for the fearful condition: Infants had significantly longer latencies to find the targets when they were primed with a mother’s fearful face, compared to a stranger’s, *F*(1, 2405.87)=4.058, *p*<0.05. Moreover, *post-hoc* analyses for the maternal prime condition, *F*(3, 2411.59)=6.54, *p*<0.001, revealed that infants were fastest to locate the target after seeing the mothers angry face, compared to seeing the mothers fearful (*p*<0.05) or happy face (*p*<0.001).

**Table 2:** Significant main effects and interactions from the LMM with participant as a random effect, emotion as a categorical fixed effect, PANAS as a continuous fixed effect, and coded familiarity and set size as continuous fixed effects.

	*df*	*F*	Value of *p*
Set Size	2431.46	2321.26	0.000
Emotion	2405.98	5.46	0.001
Emotion×Set Size	2402.59	6.10	0.000
Emotion×Familiarity	2395.89	3.00	0.029
Emotion×Set Size×PANAS	2400.75	4.07	0.007

*The dependent measure was defined as latency in milliseconds to locate the target in the visual search*.

**Figure 4:**
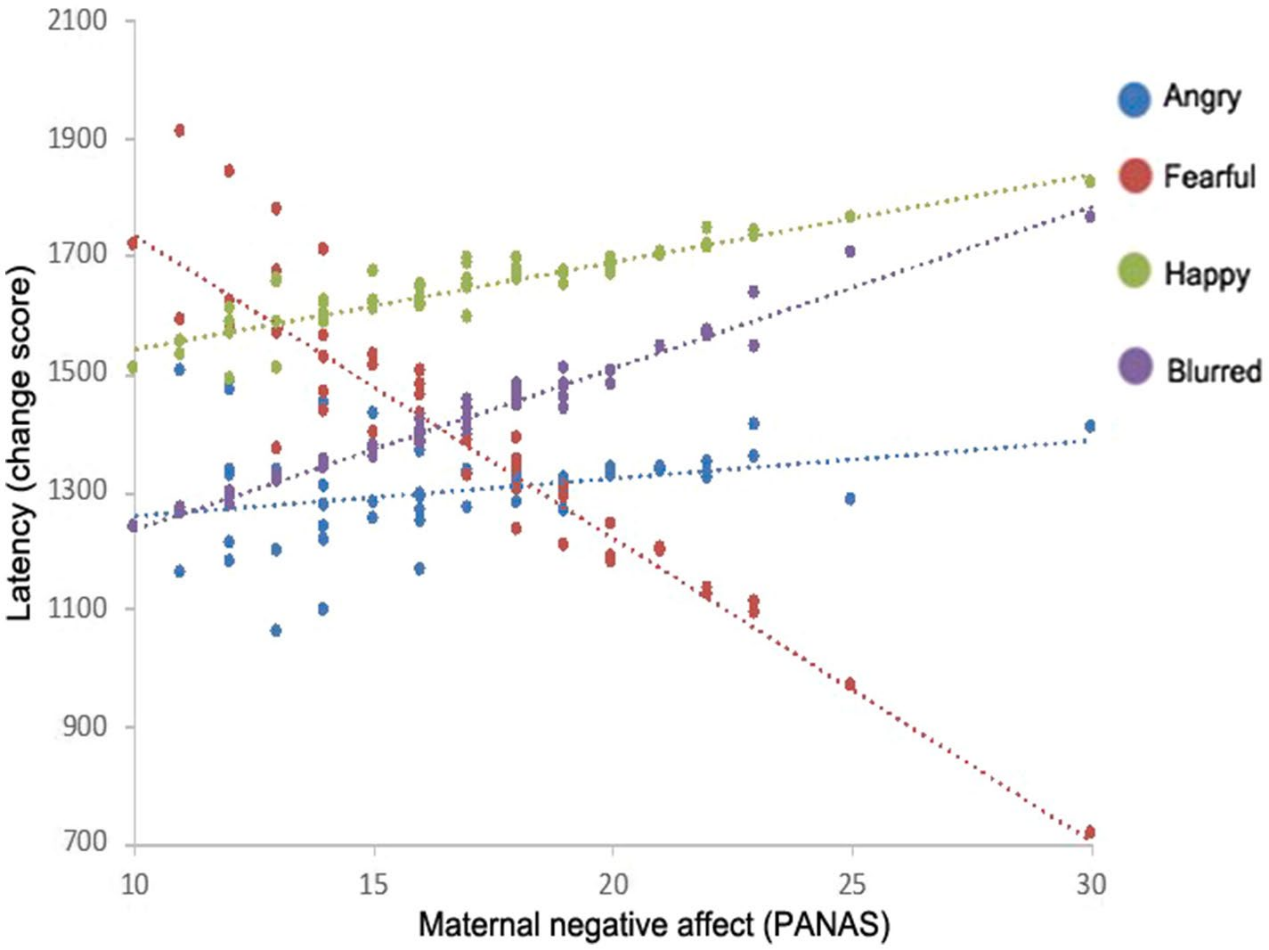
Interactions between maternal negative affect and the latency to find the target on the visual search task for each face emotion.

Furthermore, model results indicated significant interactions with infant’s context, infant’s immediate context, and visual attention difficulty. An interaction between emotion and set size revealed that there was a main effect of emotion for the set size 5 condition only (see Figure 3B); however, this was superseded by a final three-way interaction of emotion, maternal negative affect, and set size. Parameter estimates indicated that for the fearful condition subset of the set size 5 condition, latency to detect the target varied as a function of maternal negative affect, *t*(121.46)=−2.92, *p*<0.001 (see Figure 4). Specifically, for the fearful condition subset of the set size 5 condition, infants with mothers who scored low on negative affect demonstrated the slowest latencies to detect the target, while infants of mothers with high negative affect scores had the shortest latencies to detect the targets.

## Discussion

We examined 9-month-old infants’ performance on a visual search after being primed with an emotional face, in relation to their mothers’ levels of maternal affect, and whether the primed face was the mother or a stranger’s face. We predicted that the negative affect of the mother would interact with the face emotion primes in the immediate context and result in modulations of selective visual attention. Across all face types, infants showed a faster search time after being primed with an angry, fearful, or blurred face as compared to a happy face. However, when considering whether the face was that of the infants’ mother or a stranger, infants showed faster search times if the angry face was their own mother’s face, but faster search times if the fearful face was that of a stranger. If the mother had high negative affect, then infants were even faster on the visual search task after being exposed to a fearful face.

The results of the present study demonstrate for the first time that maternal negative affect and face primes in an immediate context have the capacity to jointly impact real-world subsequent visual attention. It is clear that a multiple contexts modulate selective attentional mechanisms, both as a result of an immediate stimulus (the emotion of a face) and as a result of more general aspects of the infant’s maternal environment, such as maternal affect and face familiarity. Faces communicate important social information, and as observed in adults, facial emotion information plays a functional role in guiding infants’ distribution of attention in their environment. Specifically, 9-month-old infants in the current study demonstrated faster visual search times on a non-emotionally valenced search task after being primed with an angry or fearful face compared to a happy face. Furthermore, for infants of mothers with higher negative affect scores, visual search times become even faster after being primed with a fearful face.

A possible explanation for the patterns of results related to modulations as a function of fear and maternal negative affect is that infants have learned associations between emotional contexts and their environment, resulting in individual differences in attentional mechanisms becoming attuned to the contingencies of affective signals in the environment (Pollak and Sinha, 2002). Indeed, studies have shown that visual selective attentional mechanisms “learn” through experience, and that processing resources become allocated to objects, features, and locations, which are likely to optimize the infant’s interaction with the surrounding environment and maximize positive outcome (Chelazzi et al., 2013). From this perspective, infants learn affective signals in their environment over time, and different emotional contexts result in differences in how the infant attends to their environment. We know that exposure to mothers’ emotional expression of fear plays a role in the development of infants’ attention to facial expressions in typical development (Aktar et al., 2018). For example, infants of mothers with higher negative affect have shown an increase in visual attention toward fearful faces, even when controlling for novelty of the face (Peltola et al., 2009). Those infants that are regularly in an environment with higher negative affect likely have increased exposure to negative emotions, such as fear, resulting in a disruption in the perceptual representations of basic emotions, such as a higher sensitivity to negative emotions (Pollak and Kistler, 2002; Pollak and Sinha, 2002). It is therefore plausible that these changes in attentional mechanisms are adaptive in serving to increase vigilance in the environment. While the happy face did result in overall slower latencies, the present findings support an adaptive perspective on attentional mechanisms that is supported by the additive effect of maternal negative affect on infants’ visual attention, demonstrating flexibility and adaption to the infants’ affective environment.

Fear, in the context of both emotional processing and the relational context, is an emotion with a particularly powerful impactful on infants’ attention regardless of face familiarity. In the current study, there was an interaction between fear expressions and maternal negative affect that resulted in infants’ increased vigilance in the visual field and faster search times. Work in adults utilizing similar paradigms has similarly found fear-related modulations in selective attention as function of priming (Becker, 2009, Haas et al., 2017). Prior work has suggested that ambiguity of fearful faces drives these fear-related effects: In order to identify the locus of threat, fast disengagement from the fearful face is necessary to search ones environment (Peltola et al., 2009).

The current study and the findings presented here are relatively new in infant research, and we based the research questions in part on adult literature in visual attention. We find that for infants, the happy faces resulted in slower visual search than the negative faces. Our interpretation, which is consistent with adult literature, is that after viewing a happy face, it is likely that there is no vigilance required in infants’ visuals search (no inhibition in their visual exploration) and is associated with more global processing. In contrast, when negatively primed, infants have more hypervigilance (Marshall et al., 2009; Richards et al., 2014). It is interesting that these patterns in infancy are similar to what has been shown in adults. Studies have shown that viewing images that imply threat, such as fearful faces, results in a modulations in attention (also referred to as “weapon focus”; Christianson, 1992). Attention becomes hyper-vigilant and optimized to detect threat. There are indeed benefits to this focused mode of attention, but it also comes at a cost. Other less critical aspects of the experience, such as details in the environment or of persons, are attended too less carefully, resulting in limitations to the global encoding of information. This is also why we see the effects in the current study limited to set size 5, when the distractor images must be inhibited during focused visual search.

Anger was also a particularly salient emotional prime for infants in this study, but only for the mother’s face and not for a stranger’s, although this effect only approached statistical significance (*p*=0.051). Indeed, the finding that angry faces facilitated attention to the targets is consistent with previous research on rapid threat detection. Although a familiarity effect for the angry face was only a trend, it still might suggest that a mother’s emotionally angry face may uniquely important for infants at this stage of development, similar to the fearful face. One possible explanation for the significant familiarity effect of fear and the trend for anger is found in prior work showing that infants with mothers that have symptoms of anxiety have a specific visual sensitivity to negative emotional expressions (Kataja et al., 2019). The trending difference between mothers and strangers could reflect a possible familiarity effect to particular emotions from infants’ mothers, such as less familiarly to angry expressions by their mothers, while fear provides a hypervigilance. This novelty to an angry mother’s face could be particularly strong due to infants’ attunement to their mothers.

Research exploring children’s social referencing suggests that infants draw on emotional information from their mother (Mumme et al., 1996), and even differentiate between mothers and strangers (Stenberg, 2009; Stenberg and Forslund, 2020). For example, Kataja et al. (2019) showed that maternal anxiety was associated with a faster disengagement from the mothers’ angry faces. Other work has shown that a mother’s overemphasis on negative emotions, such as anger, can interfere with the mother’s capacity to accurately read the infant’s emotional state and regulatory needs (Rosenblum et al., 2006). Such an environment has been shown to disrupt the development of age-appropriate attentional mechanisms and lead to either an increased sensitivity to negative emotions in children from aversive environments (Pollak and Kistler, 2002; Pollak and Sinha, 2002) or a general decrease in the ability to process and identify emotional facial expressions during childhood (Forslund et al., 2017; Gredebäck, et al., 2021). We know that infants are expert face processors toward their primary caregiver’s face due to their reliance in the first year of life on facial information and cues, such as emotional expression and eye gaze (Posner et al., 2014; Rennels et al., 2017). This prior work is consistent with our findings and supports a differentiation between a familiar caregiver’s face and a stranger’s, depending on emotion, and that this information differentially impacts subsequent visual attention. Familiarity and expertise with their mother’s face, and familiarity with particular emotional expressions displayed by their mothers, may be driving this difference.

There are different possible explanations for the interaction between negative affect and emotion on infants’ visual attention. It is possible that negative affect measured in the current study reflects transient effects (e.g., maybe the mothers just had a bad week). However, it is worth noting that the findings presented here are in line with previous studies (Marshall et al., 2009; Richards et al., 2014). We suggest that these findings provide further evidence that changes in attentional mechanisms are adaptive in serving to increase vigilance in the environment and are parsimonious with the prior literature. Maternal negative affect is commonly conceptualized as a mood disturbance (Hanley et al., 2014), but this may include anything from increased exposure to negative facial expressions, less exposure to positive facial expressions, negative vocal expressions, less interaction overall, or a higher exposure to conflict. The current study cannot differentiate among these particular effects but does suggest a general measure of negative affect is significantly important for infants’ selective visual attention. Further research should explore what it is specifically about negative affect that may be influencing infants’ visual attention.

In both the context of maternal affect and immediate facial expressions, it is possible that if an infant is regularly exposed to negative emotions, their visual selective attention may be in a constant state of arousal and heightened sensitivity. Although the current study cannot distinguish between transient and long-term effects, considering how possible long-term implications of negative affect and emotional context might vary over time are warranted and important to explore in future studies. Future studies should examine possible implications related to attachment theory, consequences for caregiver-child relationships, and social and emotional development.

## Conclusion

The current study demonstrates that infants’ visual selective attention mechanisms are modulated by exposure to a facial emotion and by their mothers’ negative affect. There are different perspectives that can account for these findings, such as an innate threat bias mechanism (Kataja et al., 2019, 2020) or long-term effects from experiential processes in perceptual learning, such as in negative affective environments (Pollak and Sinha, 2002). Data from the present study are not sufficient to identify the precise mechanism behind face emotion and visual attention, but it does seems likely that infants develop attentional mechanisms that are sensitive to their affective experiences (Pollak and Sinha, 2002) and that these attentional mechanisms become adjusted to better adapt to their environment. We only examined infants’ visual attention in the short term, so we can only speculate how these findings might generalize over a long-term period. It may be that changes to infants’ visual attention are only a brief effect. However, if the emotional contexts measured here are systematic factors within the infant’s environment, then the effects on infants’ visual attention system are reoccurring on a regular basis and may potentially have long-term consequences on developmental processes.

We believe the current study opens many paths for future studies and further research. Since this is one of the first studies to examine selective visual attention and the joint impact of multiple emotional contexts of the child, we still do not know to what extent selective visual attention might serve the same functionality in infancy as adulthood. It is very possible that changes to visual attention, perhaps as a result of maladaptive consequences, may have long-term consequences. To fully understand how environment and emotional context of the child in the first year impacts infant attention throughout development, a longitudinal study would be needed. Indeed, this study is only a snapshot of development and therefore cannot make assumptions regarding change over time or make qualitative assessments about what constitutes good vs. bad adaptive behavior.

The results provide compelling evidence for the functional role of emotional contexts, such as maternal affect and seeing a fearful face, modulating infants’ selective visual attention within their immediate environment. Both the immediate emotional face primes and maternal affect impacted infants’ performance during a visual search, demonstrating their capacity to influence what infants attend to and subsequently process in their environment. What is still unknown is how this actually might affect infant’s goal-directed behaviors, learning, and other developmental outcomes. Future studies are needed to address the potential impact on these learning processes in the short and long terms.

## Data Availability Statement

The raw data supporting the conclusions of this article will be made available by the authors, without undue reservation.

## Ethics Statement

The studies involving human participants were reviewed and approved by the Swedish Ethical Review (Etikprövningsmyndigheten). Written informed consent to participate in this study was provided by the participants’ legal guardian/next of kin.

## Author Contributions

JJ contributed to conception, design, data collection, and writing. SH and GG contributed to conception, design, and writing. NF contributed to design and writing. All authors contributed to the article and approved the submitted version.

## Conflict of Interest

The authors declare that the research was conducted in the absence of any commercial or financial relationships that could be construed as a potential conflict of interest.

## Publisher’s Note

All claims expressed in this article are solely those of the authors and do not necessarily represent those of their affiliated organizations, or those of the publisher, the editors and the reviewers. Any product that may be evaluated in this article, or claim that may be made by its manufacturer, is not guaranteed or endorsed by the publisher.

## Supplementary Material

The Supplementary Material for this article can be found online at: https://www.frontiersin.org/articles/10.3389/fpsyg.2021.700272/full#supplementary-material
