# Supplementary material for: Infants’ Selective Visual Attention Is Dependent on Maternal Affect and Emotional Context
Source: Front Psychol. 2021 Sep 16;12:700272. doi: 10.3389/fpsyg.2021.700272 (PMC8481686; doi:10.3389/fpsyg.2021.700272)
Supplement: Supplementary file 1 [file Table_1.DOCX]

**Supplementary Materials**

**Infant Temperament**

**The Infant Behavior Questionnaire-Revised (IBQ-R).** The aim of the study was to investigate an integrated impact of caregivers with high levels of negative affect, exposure to negative emotional expressions, as well as certain temperamental traits of infants. To measure infant temperament, we used the very short version of The Infant Behavior Questionnaire—Revised (IBQ-R; Gartstein & Rothbart, 2003) is a parent-report measure of infant temperament consisting of 36 items with 3 broad scales (surgency, negative affectivity, regulatory capacity). Reliability and validity of the IBQ-R has been supported for samples from multiple cultures, with Cronbach's alpha's ranging from 0.77 to 0.96 (Gartstein & Rothbart, 2003; Gartstein, Knyazev, & Slobodskaya, 2005). We used the officially translated Swedish language version for the current study (Gartstein & Rothbart, 2003).

**Data analysis.** To first assess multicollinearity assumptions, we examined the correlation between maternal negative affect and infant negative affect, yielding a Pearson *r* = .21, *p* < .001. Given the high correlation and concerns of reliability of maternal report of infant behavior, only maternal self-report on the PANAS was examined in the final LMM model. The decision to rely on the PANAS rather than the IBQ-R was due to open questions in the literature regarding reliability and validity of parent-reported temperament questionnaires (Seifer, 2002).

**Results**

**Table S1.** *The full LMM with participant as a random effect, emotion (angry, fearful, happy, blurred) as a categorical fixed effect, maternal negative affect (PANAS) as a continuous fixed effect, as well as coded familiarity (mother = 0, stranger = 1), and set size type (no distractors = 0, 4 distractors = 1) as continuous fixed effects. The dependent measure was defined as latency in milliseconds to locate the target in the visual search.*

|  | | | |  |  |
| --- | --- | --- | --- | --- | --- |
| Source | Num.df | Den. df | F | Sig. |  |
| Intercept | 1 | 79.720 | 5756.946 | .000 |  |
| SetSize | 1 | 2431.458 | 2321.264 | .000 |  |
| Emotion | 3 | 2405.975 | 5.456 | .001 |  |
| PANAS | 1 | 79.498 | .015 | .902 |  |
| Familiarity | 1 | 2395.018 | .102 | .750 |  |
| Emotion*PANAS | 3 | 2402.772 | 2.407 | .065 |  |
| Emotion*SetSize | 3 | 2402.591 | 6.095 | .000 |  |
| Emotion*Familiarity | 3 | 2395.886 | 3.004 | .029 |  |
| SetSize*PANAS | 1 | 2424.116 | .014 | .907 |  |
| Familiarity*PANAS | 1 | 2399.849 | .240 | .625 |  |
| SetSize*Familiarity | 1 | 2381.685 | .258 | .612 |  |
| Emotion*SetSize*PANAS | 3 | 2400.752 | 4.068 | .007 |  |
| Emotion*Familiarity*PANAS | 3 | 2398.363 | 1.313 | .268 |  |
| Emotion*SetSize*Familiarity | 3 | 2406.255 | 2.357 | .070 |  |
| SetSize*Familiarity*PANAS | 1 | 2393.869 | .083 | .773 |  |
| Emotion*SetSize*Familiarity*PANAS | 3 | 2405.790 | 2.015 | .110 |  |

**Table S2.** *The parameter estimates for the results from the LMM.*

|  | | | | | | | |
| --- | --- | --- | --- | --- | --- | --- | --- |
| Parameter | Estimate | Std. Error | df | t | Sig. | 95% CI - | 95% CI + |
|  |  |  |  |  |  |  |  |
| Angry | 16.10 | 65.91 | 2,383.27 | 0.24 | 0.81 | -113.14 | 145.35 |
| Fearful | 18.93 | 66.56 | 2,387.33 | 0.28 | 0.78 | -111.59 | 149.45 |
| Happy | 28.46 | 65.93 | 2,392.65 | 0.43 | 0.67 | -100.83 | 157.75 |
| Blurred | 583.81 | 47.26 | 1,770.11 | 12.35 | 0.00 | 491.11 | 676.51 |
|  |  |  |  |  |  |  |  |
| Angry*PANAS | -1.30 | 64.66 | 2,380.40 | -0.02 | 0.98 | -128.10 | 125.50 |
| Fearful*PANAS | 42.11 | 67.45 | 2,383.78 | 0.62 | 0.53 | -90.16 | 174.38 |
| Happy*PANAS | 34.90 | 64.59 | 2,392.80 | 0.54 | 0.59 | -91.76 | 161.55 |
| Blurred*PANAS | -34.50 | 46.19 | 1,726.91 | -0.75 | 0.46 | -125.09 | 56.10 |
|  |  |  |  |  |  |  |  |
| Angry*SetSize5 | -196.92 | 120.41 | 2,406.63 | -1.64 | 0.10 | -433.04 | 39.19 |
| Fearful*SetSize5 | 123.65 | 121.83 | 2,416.88 | 1.02 | 0.31 | -115.24 | 362.55 |
| Happy*SetSize5 | 271.31 | 116.09 | 2,415.07 | 2.34 | 0.02 | 43.66 | 498.95 |
| Blurred*SetSize5 | 1,404.20 | 84.67 | 2,418.69 | 16.58 | 0.00 | 1,238.17 | 1,570.24 |
|  |  |  |  |  |  |  |  |
| Angry*Stranger | 16.11 | 93.59 | 2,384.98 | 0.17 | 0.86 | -167.41 | 199.63 |
| Fearful*Stranger | -2.71 | 93.90 | 2,387.21 | -0.03 | 0.98 | -186.84 | 181.43 |
| Happy*Stranger | -35.67 | 94.20 | 2,389.79 | -0.38 | 0.71 | -220.40 | 149.06 |
| Blurred*Stranger | 11.16 | 66.08 | 2,389.18 | 0.17 | 0.87 | -118.42 | 140.74 |
|  |  |  |  |  |  |  |  |
| Angry*PANAS*SetSize5 | 49.44 | 117.25 | 2,402.53 | 0.42 | 0.67 | -180.48 | 279.36 |
| Fearful*PANAS*SetSize5 | -355.15 | 121.46 | 2,415.52 | -2.92 | 0.00 | -593.33 | -116.96 |
| Happy*PANAS*SetSize5 | -80.72 | 115.12 | 2,417.18 | -0.70 | 0.48 | -306.46 | 145.02 |
| Blurred*PANAS*SetSize5 | 108.74 | 84.19 | 2,417.69 | 1.29 | 0.20 | -56.36 | 273.83 |
|  |  |  |  |  |  |  |  |
| Angry*PANAS*Stranger | 18.79 | 93.39 | 2,371.20 | 0.20 | 0.84 | -164.35 | 201.92 |
| Fearful*PANAS*Stranger | -9.39 | 95.55 | 2,387.69 | -0.10 | 0.92 | -196.75 | 177.97 |
| Happy*PANAS*Stranger | -33.91 | 93.75 | 2,394.42 | -0.36 | 0.72 | -217.75 | 149.94 |
| Blurred*PANAS*Stranger | 29.43 | 66.55 | 2,391.71 | 0.44 | 0.66 | -101.07 | 159.92 |
|  |  |  |  |  |  |  |  |
| Angry*SetSize5*Stranger | 146.04 | 168.09 | 2,375.79 | 0.87 | 0.39 | -183.57 | 475.65 |
| Fearful*SetSize5*Stranger | -295.17 | 169.76 | 2,408.47 | -1.74 | 0.08 | -628.06 | 37.72 |
| Happy*SetSize5*Stranger | -101.76 | 165.77 | 2,400.63 | -0.61 | 0.54 | -426.82 | 223.29 |
| Blurred*SetSize5*Stranger | 32.53 | 118.03 | 2,404.05 | 0.28 | 0.78 | -198.92 | 263.97 |
|  |  |  |  |  |  |  |  |
| Angry*PANAS*SetSize5*Stranger | -242.99 | 165.10 | 2,377.68 | -1.47 | 0.14 | -566.75 | 80.78 |
| Fearful*PANAS*SetSize5*Stranger | 140.77 | 175.14 | 2,410.43 | 0.80 | 0.42 | -202.67 | 484.20 |
| Happy*PANAS*SetSize5*Stranger | 68.25 | 168.09 | 2,403.97 | 0.41 | 0.69 | -261.37 | 397.87 |
| Blurred*PANAS*SetSize5*Stranger | -8.78 | 120.02 | 2,412.47 | -0.07 | 0.94 | -244.13 | 226.58 |

References

Gartstein, M. A., & Rothbart, M. K. (2003). Studying infant temperament via the revised infant behavior questionnaire. *Infant Behavior and Development, 26*(1), 64-86.

Seifer, R. (2002). What do we learn from parent reports of their children's behavior? Commentary on

Vaughn et al.'s critique of early temperament assessments. Infant Behavior & Development, 25(1), 117–120. https://doi.org/10.1016/S0163-6383(02)00110-8

Gartstein, M. A., Knyazev, G. G., & Slobodskaya, H. R. (2005). Cross-cultural differences in the structure of infant temperament: United States of America (US) and Russia. *Infant Behavior and Development, 28*(1), 54-61.
